# Dietary hemoglobin rescues young piglets from severe iron deficiency anemia: Duodenal expression profile of genes involved in heme iron absorption

**DOI:** 10.1371/journal.pone.0181117

**Published:** 2017-07-13

**Authors:** Robert Staroń, Paweł Lipiński, Małgorzata Lenartowicz, Aleksandra Bednarz, Anna Gajowiak, Ewa Smuda, Wojciech Krzeptowski, Marek Pieszka, Tamara Korolonek, Iqbal Hamza, Dorine W. Swinkels, Rachel P. L. Van Swelm, Rafał R. Starzyński

**Affiliations:** 1 Institute of Genetics and Animal Breeding PAS, Department of Molecular Biology, Jastrzębiec, Poland; 2 Department of Genetics and Evolution, Institute of Zoology, Jagiellonian University, Kraków, Poland; 3 Department of Animal Nutrition & Feed Science, National Research Institute of Animal Production, Kraków, Poland; 4 Department of Animal and Avian Sciences and Department of Cell Biology and Molecular Genetics, University of Maryland, College Park, Maryland, United States of America; 5 Department of Laboratory Medicine (LGEM 830), Radboud University Medical Centre, Nijmegen, The Netherlands; Lady Davis Institute for Medical Research, CANADA

## Abstract

Heme is an efficient source of iron in the diet, and heme preparations are used to prevent and cure iron deficiency anemia in humans and animals. However, the molecular mechanisms responsible for heme absorption remain only partially characterized. Here, we employed young iron-deficient piglets as a convenient animal model to determine the efficacy of oral heme iron supplementation and investigate the pathways of heme iron absorption. The use of bovine hemoglobin as a dietary source of heme iron was found to efficiently counteract the development of iron deficiency anemia in piglets, although it did not fully rebalance their iron status. Our results revealed a concerted increase in the expression of genes responsible for apical and basolateral heme transport in the duodenum of piglets fed a heme-enriched diet. In these animals the catalytic activity of heme oxygenase 1 contributed to the release of elemental iron from the protoporphyrin ring of heme within enterocytes, which may then be transported by the strongly expressed ferroportin across the basolateral membrane to the circulation. We hypothesize that the well-recognized high bioavailability of heme iron may depend on a split pathway mediating the transport of heme-derived elemental iron and intact heme from the interior of duodenal enterocytes to the bloodstream.

## Introduction

Heme, a ferrous iron protoporphyin IX complex, is employed as a prosthetic group in diverse proteins that participate in important biological processes [1]. The provision of an adequate amount of iron for heme biosynthesis is essential for intracellular iron homeostasis. Likewise, the delivery of iron for heme/hemoglobin synthesis in erythroblasts is indispensable for maintenance of the body iron balance. These processes rely on the recovery of iron from senescent erythrocytes, through the circulation. Molecular coordination of these activities involves the functions of heme oxygenase 1 (HO1), an inducible heme-degrading enzyme [2], the post-transcriptional IRE/IRP system [3], as well as the hepcidin-ferroportin regulatory axis [4]. Interestingly, recent mammalian studies have demonstrated the existence of an expanded system of proteins involved in the transport of intact heme across biological membranes [5–8]. Heme exported to the bloodstream is scavenged by hemopexin (Hpx), an effective heme-binding protein found in blood plasma, which acts primarily to deliver heme to cells *via* CD91 receptor-mediated endocytosis [9,10]. This system is of particular importance when the concentration of free heme reaches toxic levels in the body [5] or locally in cells with an intensive heme metabolism [11]. On the other hand, there is growing evidence that the movement of intact heme molecules takes place under physiological conditions and constitutes an integral part of iron turnover in the body [12]. It has long been known that exogenous heme can be efficiently taken up by enterocytes as an intact metalloporphyrin molecule *via* receptor-mediated endocytosis, and thus may provide a highly bioavailable source of dietary iron for the organism [13–15]. Although recent studies have led to the identification of intestinal heme transporters [16,17], the molecular mechanisms of dietary heme absorption remain the subject of some controversy [15,18] and our understanding of this process is far from complete.

The objectives of this study were to test the efficacy of dietary hemoglobin-derived heme iron in rectifying iron deficiency anemia (IDA) and to identify the molecular pathways of heme iron trafficking across duodenal enterocytes. We used iron-deficient piglets in our experiments because evidence from our previous studies indicated that in the neonatal period, piglets represent a valuable animal model for testing strategies for supplementation with exogenous iron [19–22]. The existence of a brush-border heme receptor in the pig intestine reported in the 1970s/1980s [23,24] was an additional reason for choosing this animal model for our study. Finally, since the bioavailability of heme is greater under conditions of iron deficiency [25], it was thought that IDA in piglets would be useful in deciphering the pathways of dietary heme absorption.

We show that oral supplementation of piglets with hemoglobin rescues them from severe IDA observed in non-supplemented animals. The expression of genes responsible for apical and basolateral heme transport, and heme breakdown was increased in the duodenum of piglets receiving a hemoglobin-enriched diet. In addition, we demonstrate that the hepcidin-duodenal ferroportin axis acts to enhance the basolateral transport of elemental iron released from heme in enterocytes. We hypothesize that the well-known high bioavailability of heme iron may rely on the presence of two independent pathways mediating the transport of heme-derived elemental iron and intact heme from the duodenal enterocytes into the circulation.

## Results

### Oral hemoglobin supplementation rescues piglets from severe IDA and maintains their growth performance

Supplementation of the diet of piglets with hemoglobin efficiently prevented the deterioration of their hematological indices and plasma iron levels, and rescued them from the severe anemia observed in non-supplemented animals (Table 1). Piglets receiving bovine hemoglobin orally maintained their blood hemoglobin level at around the threshold value for anemia in pigs, i.e. 8g/dL [26], throughout the experimental period (Table 1). Piglets receiving high levels of FeDex by intramuscular injection showed significantly higher hematological and plasma iron performance, excluding the RBC count (Table 1). The main reason for the greater efficacy of this routine iron therapy is the direct delivery of a much higher amount of iron. Considering that the total intake of iron in feed containing hemoglobin was about 120mg (S1 Table), and assuming that the maximal rate of heme iron absorption in mammals is around 50% [13], we estimate that piglets in this group assimilated about 70 mg Fe between the 3^rd^ and 28^th^ day after birth. The intake of iron was particularly low between days 3 and 9 due to the poor consumption of feed by pig neonates during this period (S1 Table). Therefore, a diet enriched in hemoglobin becomes a significant source of bioavailable dietary iron only from the second week of life, when feed intake by piglets increases. Piglets receiving hemoglobin *per os* tended to show a greater b.w. gain during the first 4 weeks of life, similarly to those given FeDex injections (S2 Table). A split supplementation consisting of the injection of a small amount of FeDex on day 3 after birth plus oral supplementation with hemoglobin resulted in a better hematological status of piglets (Table 1).

**Table 1:** Hematological parameters and plasma iron concentration (mean ± S.D.) of experimental piglets.

	RBC (x 10^6^/μL)				HGB (g/dL)				HCT (%)				Plasma iron level (μM/L)			
Age (Days)Group	3	14	21	28	3	14	21	28	3	14	21	28	3	14	21	28
**Control**	a**3.9**±0.1	**4.3**±0.6	**4.7**±0.6	a**4.9**±0.6	A**8.0**±0.4	A**6.4**±0.8	A**6.2**±0.5	A**5.7**±0.8	Aa**27.0**±1.2	a**23.0**±3.0	A**21.3**±1.5	A**21.1**±2.2	A**8.8**±1.2	A**2.8**±1.5	A**2.6**±1.5	A**1.6**±0.5
**Iron dextran**	A**4.5**±0.2	A***5.9±0.3	A***6.4±0.5	A***6.6±0.5	A**8.5**±0.7	A***11.8±0.6	A***°°**12.3**±0.8	A***°°**12.4**±0.6	A**27.8**±2.4	A***°°**37.7**±1.7	A***°°**37.3**±2.2	A***°°**37.7**±1.9	A**7.8**±2,5	A***°°**27.8**±3.6	A***°°**28.1**±3.9	A***°°**30.3**±3.5
**Hemoglobin**	A**4.5**±0.3	**5.6±0.3	*^5.7±0.5	A**6.3±0.6	**8.3**±0.5	***^^8.6±0.7	**^^**°°**7.5**±1.0	**°°**7.7**±0.7	**28.7**±2.0	**^^°°**29.5**±2.2	*^°°**26.4**±3.6	**°°**26.5**±3.4	**5.3**±2.7	**^^**°°**1.4**±1.2	°°**4.4**±1.6	**°°**10.8**±7.4
**Iron dextran +Hemoglobin**	A**4.7±**1.0	A***6.1±0.4	A***^6.5±0.3	A***6.8±0.6	**9.1±**1.8	***^^10.5±0.9	***^^°°**9.9±**0.6	***°°**9.1±**0.8	**29.9±**5.5	***^^35.5±2.9	***^°°**32.1±**2.0	***°°**30.6±**2.3	**8.4±**4.6	**^^°°**12.4±**3.2	***°°**9.3±**2.3	**°°**9.9±**4.5

RBC–red blood cell count, HGB–hemoglobin level, HCT–hematocrit. All parameters were determined for 15 piglets from each experimental group except iron dextran-supplemented piglets (n = 9).

*, ** and *** asterisks denote statistically significant differences at P<0.05, P<0.01 and P<0,001 respectively, between parameters in control and other experimental groups of piglets in a given age of piglets.

Letters a and A denote significant differences at P<0.05, P<0.01 respectively, across piglet age in each group.

Symbols ^ and ^^ denote significant differences at P<0.05 and P<0,01 respectively, between parameters in Hemoglobin group *vs* Iron dextran\Hemoglobin. Symbols

° and °° denote significant differences at P<0.05 P<0,01 respectively, between parameters in groups Dextran *vs*. Hemoglobin and Dextran *vs* Iron Dextran\Hemoglobin.

*Control*, *iron dextran* and *hemoglobin* groups are described under Materials and methods. Piglets form *iron dextran + hemoglobin* group were intramuscularly injected with FeDex (40 mg Fe/kg b.w.) on day 3 after birth and fed Prestarter Wigor 1 Plus feed enriched with bovine hemoglobin (Bovogen, East Keilor, Australia) from day 3 to day 28 after birth.

### Negligible expression of divalent metal transporter (DMT1) in hemoglobin-supplemented piglets

**Fig 1:**
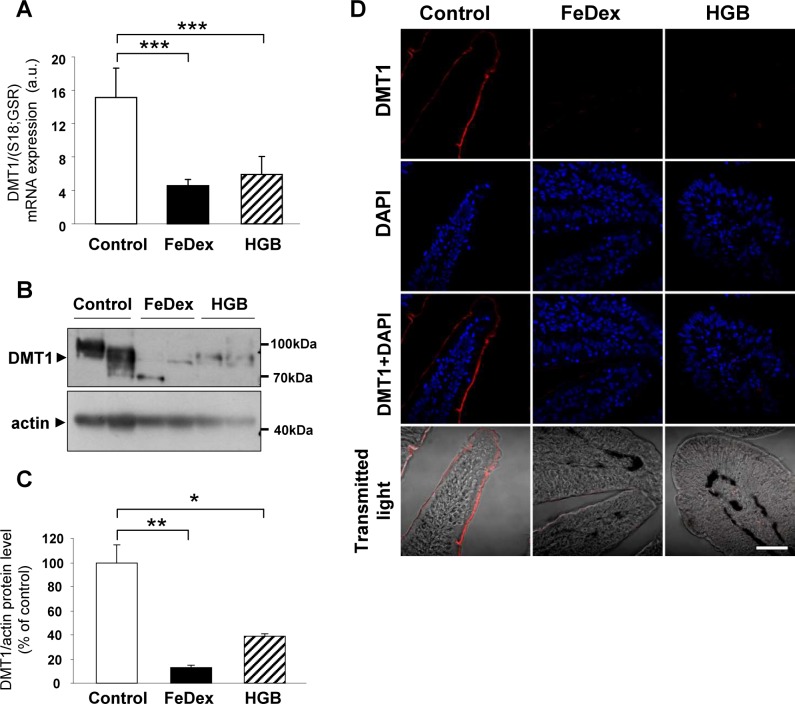
Reduced expression of DMT1 in piglets supplemented with FeDex and fed a hemoglobin (HGB)-enriched diet. **(A)** RT-qPCR analysis of DMT1 mRNA expression. The histogram displays DMT1 mRNA levels in arbitrary units (means ± S.D., n = 7). **(B and C)** Western blot analysis of DMT1 protein levels in membrane fractions prepared from duodenal scrapings. A representative immunoblot is shown **(B)**. Immunolabelled DMT1 bands from separate blots performed on scrapings isolated from 6 piglets were quantified using a Molecular Imager, and DMT1 protein levels (means ± S.D.) are plotted in arbitrary units **(C)**. **(D)** Immunofluorescent staining of DMT1 in the duodenum. To confirm the specificity of DMT1 detection, piglet duodenum sections were incubated with only the secondary antibody. No DMT1 staining was detected in these negative controls. Counterstaining of nuclei was performed with DAPI. Duodenum morphology is shown in transmitted light.

High expression of DMT1 (Slc11a2) in epithelial cells of the duodenal villi is commonly considered a hallmark of a high demand for iron to cater for the iron requirements of iron-deficient subjects [27,28]. On the other hand, local iron-dependent post-transcriptional regulation of DMT1 in enterocytes may also influence its level [29]. In anemic piglets, the expression of the *Slc11a*2 gene at both the mRNA (Fig 1A) and protein (Fig 1B and 1C) levels was found to be high. Consequently, strong DMT1 immunostaining was observed at the brush border of epithelial cells (Fig 1D). In contrast to these iron-deficient piglets, both hemoglobin- and FeDex-supplemented animals showed significantly decreased DMT1 transcript abundance (Fig 1A), with negligible levels of DMT1 protein (Fig 1B and 1C) and no DMT1 immunostaining on duodenum sections (Fig 1D).

### Increased expression of heme apical importers on duodenal enterocytes in hemoglobin-supplemented piglets

**Fig 2:**
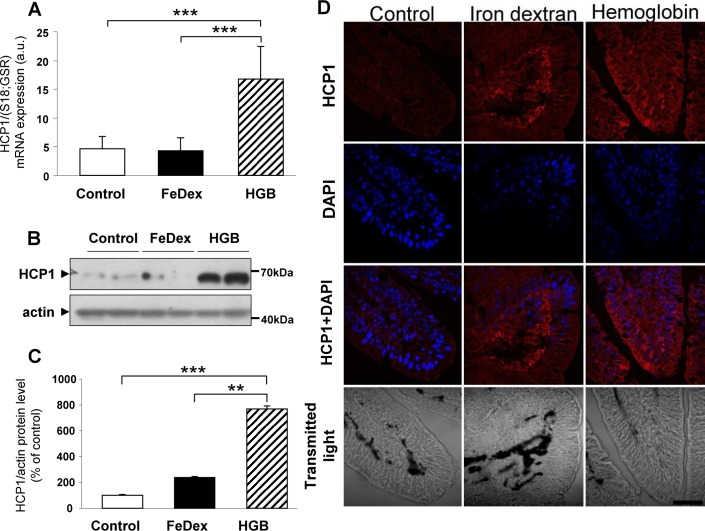
Increased HCP1 expression on the apical membrane of absorptive enterocytes of piglets fed a hemoglobin-enriched diet. **(A)** RT-qPCR analysis of HCP1 mRNA expression. The histogram displays HCP1 mRNA levels in arbitrary units (means ± S.D., n = 7). **(B and C)** Western blot analysis of HCP1 protein levels in membrane fractions prepared from duodenal scrapings. A representative immunoblot is shown **(B)**. Immunolabelled HCP1 bands from separate blots performed on scrapings isolated from 6 piglets were quantified using a Molecular Imager, and HCP1 protein levels (means ± S.D.) are plotted in arbitrary units **(C)**. **(D)** Immunofluorescent staining of HCP1 in the duodenum. To confirm the specificity of HCP1 detection, piglet duodenum sections were incubated with only the secondary antibody. No HCP1 staining was detected in these negative controls. Counterstaining of nuclei was performed with DAPI. Duodenum morphology is shown in transmitted light.

In mice the heme carrier protein 1 (HCP1/Slc46a1) has been shown to be responsible for the entry of dietary heme into enterocytes [17]. We analyzed the expression of the *Slc46a1* gene in the proximal duodenum of piglets and found that the HCP1 mRNA was up-regulated 4-fold in duodenal enterocytes from hemoglobin-supplemented animals compared with those of control and FeDex-injected piglets (Fig 2A). In addition, we demonstrated an even greater increase in the HCP1 protein level in duodenal scrapings (Fig 2B and 2C). Immunofluorescence (IF) analysis showed that HCP1 is highly expressed along the apical membrane of duodenal enterocytes (Fig 2D, right). In contrast, the enterocytes of anemic piglets showed negligible HCP1 immunostaining (Fig 2D, left). Interestingly, intense HCP1 staining was observed within the network of blood capillaries surrounded by the epithelial layer in FeDex-supplemented piglets (Fig 2D, center).

**Fig 3:**
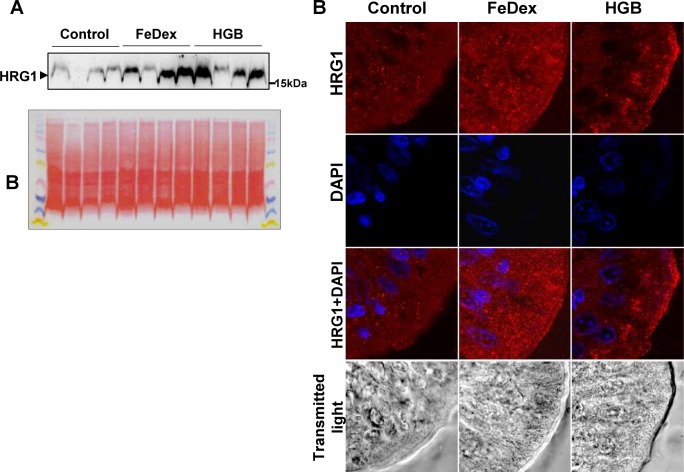
Increased HRG1 protein levels on the apical membrane of absorptive enterocytes of piglets fed a hemoglobin-enriched diet. **(A)** Western blot analysis of HRG1 protein levels in membrane fractions prepared from duodenal scrapings as described under Materials and methods. A representative immunoblot is shown. Ponceau Red staining of transferred proteins are shown (bottom panel) to confirm equivalent loading. **(B)** Immunofluorescent staining of HRG1 in the duodenum. To confirm the specificity of HRG1 detection, duodenum sections of piglets were incubated with only the secondary antibody. No HRG1 staining was detected in these negative controls. Counterstaining of nuclei was performed with DAPI. Duodenum morphology is shown in transmitted light.

Heme responsive gene 1 (HRG1/Slc48a1) has been identified as the main supplier of exogenous heme in *C*. *elegans*, a heme auxotrophic nematode [30]. Since HRG1 mRNA is abundantly expressed in cell lines derived from duodenum [30], we hypothesized that this protein may be important in the absorption of dietary heme by duodenal enterocytes of the hemoglobin-supplemented piglets. Piglets fed a hemoglobin-enriched diet as well as those injected with FeDex showed similar greatly increased levels of HRG1 compared to iron-deficient animals (Fig 3A). However, IF analysis of duodenum sections demonstrated striking differences in the localization of this protein. In piglets given hemoglobin, high levels of HRG1 were detected, mainly on the apical membrane of duodenal enterocytes (Fig 3B, right), whereas a clearly intracellular, dispersed granular/vesicular distribution of HRG1 was observed in FeDex-injected piglets (Fig 3B, center).

### Induction of HO1 expression and increased iron status in duodenal enterocytes from hemoglobin-supplemented piglets

**Fig 4:**
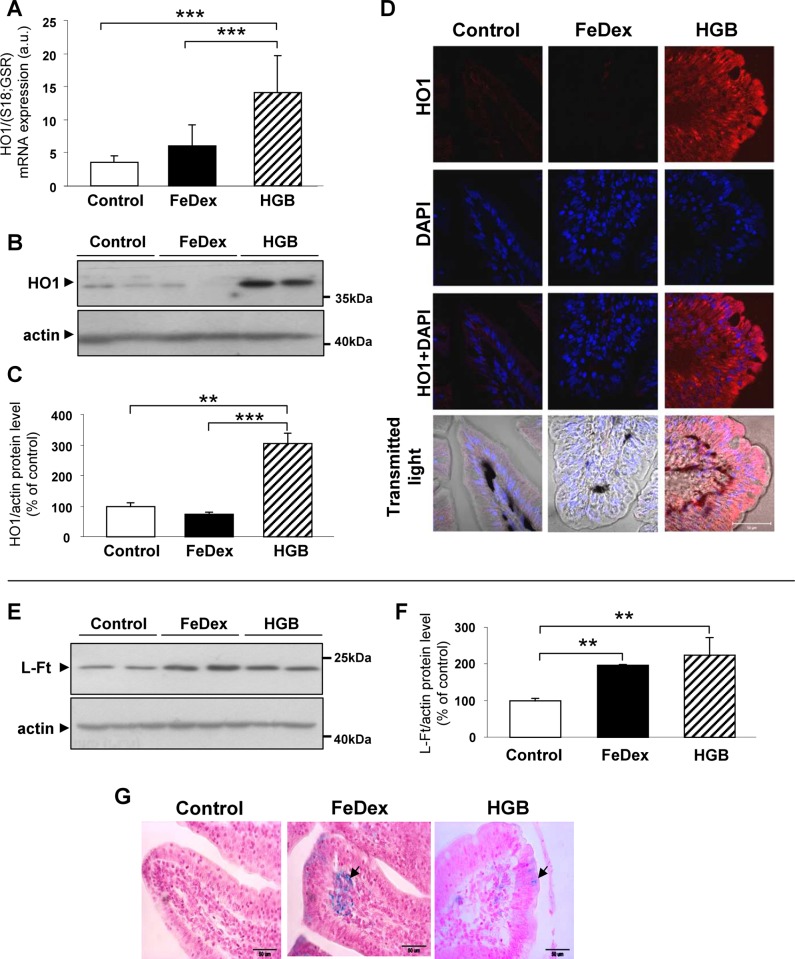
Induction of HO1 expression and increased iron status in duodenal enterocytes from hemoglobin-supplemented piglets. **(A)** RT-qPCR analysis of HO1 mRNA expression. The histogram displays HO1 mRNA levels in arbitrary units (means ± S.D., n = 7). **(B and C)** Western blot analysis of HO1 protein levels in membrane fractions prepared from duodenal scrapings. A representative immunoblot is shown **(B)**. Immunolabelled HO1 bands from separate blots performed on scrapings isolated from 6 piglets were quantified using a Molecular Imager, and HO1 protein levels (means ± S.D.) are plotted in arbitrary units **(C)**. **(D)** Immunofluorescent staining of HO1 in the duodenum. To confirm the specificity of the HO1 detection, duodenum sections of piglets were incubated with only the secondary antibody. No HO1 staining was detected in these negative controls. Counterstaining of nuclei was performed with DAPI. **(E and F)** Western blot analysis of L-ferritin protein levels in membrane fractions prepared from duodenal scrapings. A representative immunoblot is shown **(E)**. Immunolabelled L-ferritin bands from separate blots performed on scrapings isolated from 6 piglets were quantified using a Molecular Imager, and L-ferritin protein levels (means ± S.D.) are plotted in arbitrary units **(F)**. **(G)** Histological examination of iron loading in duodenum sections. Non-heme iron deposits (indicated by arrows) were detected by staining with Perls’ Prussian blue and counterstained with nuclear red. Duodenum morphology is shown in transmitted light.

HO1 (encoded by the *Hmox1* gene) is an inducible enzyme degrading heme to CO, biliverdin and ferrous ions [2]. To determine whether heme transported across the apical membrane of duodenal enterocytes triggers HO1 expression within these cells, we examined HO1 transcript and protein levels as well as HO1 distribution in the enterocytes of piglets fed a hemoglobin-enriched diet. RT-qPCR (Fig 4A) and Western blotting (Fig 4B and 4C) analyses both showed a nearly 3-fold increase in HO1 expression in these piglets compared to anemic and FeDex-supplemented animals. Microscopic analysis of transverse duodenum sections showed massive and even immunostaining of HO1 throughout the cytoplasm of duodenal enterocytes from heme-fed piglets (Fig 4D), whereas HO1 IF was barely detectable in equivalent sections from control and FeDex-injected animals. The induction of HO1 is usually coupled with an increase in the level of ferritin [31]. We found that the L-ferritin protein level was 2-fold increased in the enterocytes of hemoglobin-supplemented piglets (Fig 4E and 4F) compared to the controls, which is consistent with the presence of non-heme iron deposits detected in these cells (Fig 4G, right). In contrast, no stainable non-heme iron was detected in the duodenal enterocytes of anemic piglets (Fig 4G, left). The enterocytes of FeDex-supplemented piglets displayed L-ferritin levels that were as high as those detected in epithelial cells of the villi of hemoglobin-supplemented animals. In these iron-replete piglets, massive non-heme iron deposits were found in the capillary vessel network situated just below the epithelium of the villi (Fig 4G, center).

### High expression of ferroportin (Fpn) in the duodenum of hemoglobin-supplemented piglets is associated with a low plasma hepcidin-25 concentration

**Fig 5:**
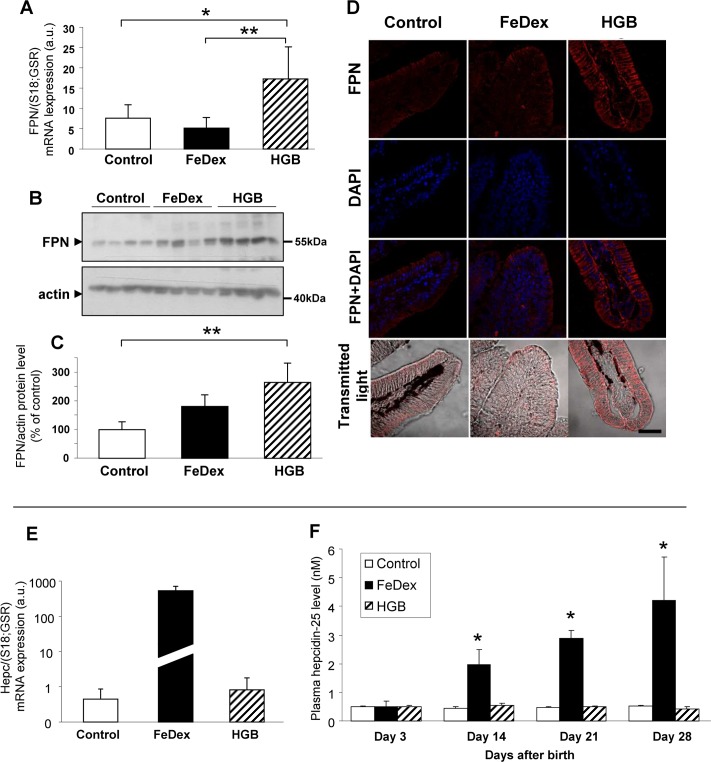
Increased Fpn expression in the duodenum of hemoglobin-supplemented piglets is associated with reductions in both hepatic hepcidin mRNA levels and plasma hepcidin-25 concentration. **(A)** RT-qPCR analysis of Fpn mRNA expression. The histogram displays Fpn mRNA levels in arbitrary units (means ± S.D., n = 7). **(B and C)** Western blot analysis of Fpn protein levels in membrane fractions prepared from duodenal scrapings. A representative immunoblot is shown **(B)**. Immunolabelled Fpn bands from separate blots performed on scrapings isolated from 8 piglets were quantified using a Molecular Imager, and Fpn protein levels (means ± S.D.) are plotted in arbitrary units **(C)**. **(D)** Immunofluorescent staining of Fpn in the duodenum. To confirm the specificity of Fpn detection, duodenum sections of piglets were incubated with only the secondary antibody. No Fpn staining was detected in these negative controls. Counterstaining of nuclei was performed with DAPI. Duodenum morphology is shown in transmitted light. **(E)** RT-qPCR analysis of hepcidin mRNA expression in the liver. The histogram displays hepcidin mRNA levels in arbitrary units (means ± S.D., n = 7). **(F)** Hepcidin concentration in the blood plasma of experimental piglets. Values are expressed as the means ± S.D. The plasma hepcidin concentration was determined for 5–7 piglets from each group/day.

Fpn (Slc40a1), localized at the basolateral membrane of duodenal enterocytes, is the major cellular non-heme iron exporter of mammals and plays a role in the transfer of duodenal iron into the circulation [32]. To study the possible involvement of Fpn in the absorption of iron released from heme within the enterocytes of hemoglobin-supplemented piglets, we examined the expression of the *Slc40a1* gene in duodenal mucosa scrapings. In the duodenum of piglets fed the hemoglobin-enriched diet, we observed a more than 2-fold increase in Fpn transcript abundance (Fig 5A) and protein level compared to control animals (Fig 5B and 5C). This upregulation was confirmed by IF analysis of duodenum tissue sections, which showed increased levels of Fpn along the basal and lateral membranes of absorptive enterocytes (Fig 5D, right). Although Fpn mRNA expression in the enterocytes of FeDex-supplemented piglets was as low as in control animals (Fig 5A), Fpn protein levels were higher, albeit not as high as in the duodenal epithelial cells of animals fed the hemoglobin-enriched diet (Fig 5B and 5C). Consequently, the intensity of Fpn immunostaining at the basolateral membrane of duodenal enterocytes of FeDex-supplemented animals was similar to that observed in the anemic piglets (Fig 5D, left). Fpn showed mainly intracellular localization in the FeDex-supplemented piglet samples (Fig 5D, center).

The amount of Fpn at the plasma membrane is tightly controlled by hepcidin-mediated regulation [4]. To determine whether duodenal Fpn is regulated by hepcidin in the experimental piglets, we analyzed expression of its mRNA in the liver and the plasma hepcidin-25 concentration in 28-day-old animals. Very low hepatic hepcidin transcript levels were detected in both the anemic and hemoglobin-supplemented piglets (Fig 5E), and these were associated with a barely detectable hepcidin-25 concentration in the blood plasma (Fig 5F). In contrast, piglets supplemented parenterally with FeDex showed highly raised levels of hepcidin mRNA and a substantially elevated hepcidin concentration in the plasma. These piglets showed a gradual increase in plasma hepcidin-25 throughout the experimental period, starting from 1nM (day 3) and then rising to more than 4nM (day 28). In comparison, the plasma hepcidin-25 concentration in control and hemoglobin-supplemented piglets remained below 1nM during the first 4 weeks of life (Fig 5F).

### Increased feline leukemia virus subgroup c receptor 1 (FLVCR1) expression on the basolateral membrane of duodenal absorptive enterocytes and decreased plasma Hpx levels in hemoglobin-supplemented piglets

**Fig 6:**
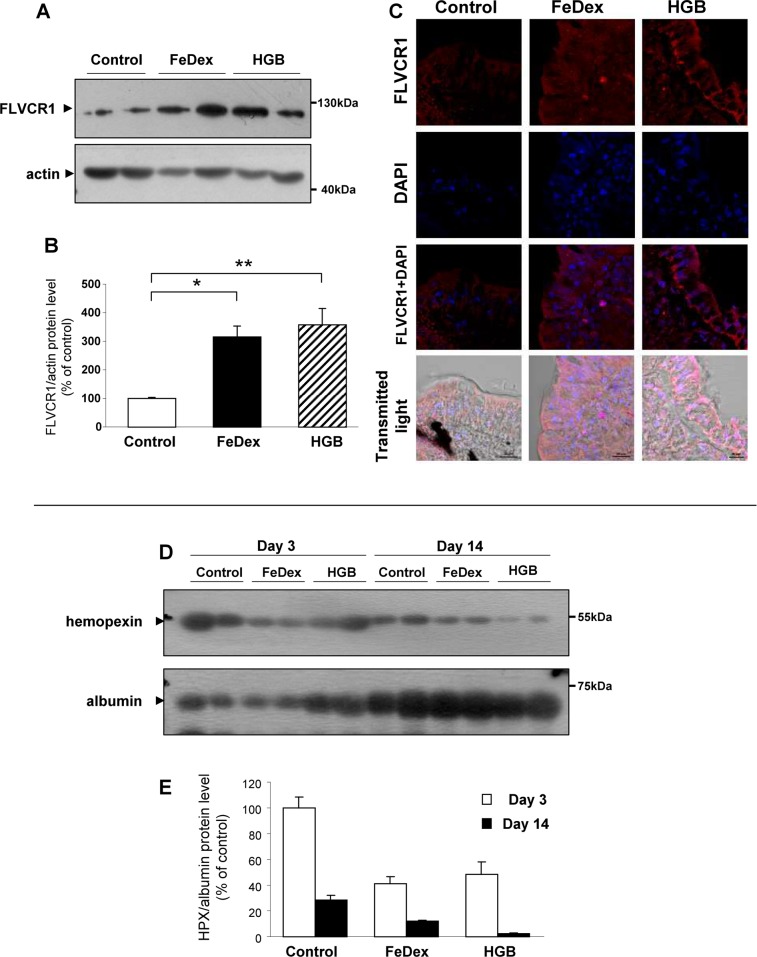
Increased FLVCR1 protein levels on the basolateral membrane of duodenal absorptive enterocytes is associated with decreased blood plasma hemopexin (Hpx) levels in hemoglobin-supplemented piglets. **(A and B)** Western blot analysis of FLVCR1 protein levels in membrane fractions prepared from duodenal scrapings. A representative immunoblot is shown **(A)**. Immunolabelled FLVCR1 bands from blots performed on scrapings isolated from 6 piglets were quantified using a Molecular Imager (Bio-Rad), and FLVCR1 protein levels (means ± S.D.) are plotted in arbitrary units **(B)**. **(C)** Immunofluorescent staining of FLVCR1 in the duodenum. To confirm the specificity of FLVCR1 detection, duodenum sections of piglets were incubated with only the secondary antibody. No FLVCR1 staining was detected in these negative controls. Counterstaining of nuclei was performed with DAPI. Duodenum morphology is shown in transmitted light. **(D and E)** Western blot analysis of Hpx levels in blood plasma. 7 *μ*l samples of 20-fold diluted piglet blood plasma were analyzed as described previously [51]. A representative immunoblot is shown **(D)**. Immunolabelled Hpx bands from separate blots performed with plasma samples collected from 6 piglets were quantified using a Molecular Imager, and Hpx protein levels (means ± S.D.) are plotted in arbitrary units **(E)**.

The mammalian heme exporter FLVCR1 (Slc49a1) is highly expressed in the small intestine. Therefore, we examined whether heme derived from dietary hemoglobin is exported *via* FLVCR1 into the circulation as an intact molecule. Western blotting of membrane extracts obtained from scrapings of the proximal portion of the duodenum of piglets fed the hemoglobin-enriched diet showed an approximately 3.5-fold increase in FLVCR1 protein level compared to control anemic piglets (Fig 6A and 6B). Similarly raised levels of FLVCR1 protein were also observed in equivalent samples from piglets injected with FeDex. IF analysis of sections obtained from the same portion of the duodenum showed strong immunostaining in piglets fed a hemoglobin-enriched diet, mainly located on the basolateral membrane of absorptive enterocytes (Fig 6C, right). In comparison, the localization of FLVCR1 in the FeDex-injected animals was mostly intracellular (Fig 6D,center). It is known that the export of heme by FLVCR1 depends on the availability of Hpx [12]. Under conditions of increased flux of heme into the circulation, serum levels of Hpx are usually low [10]. Therefore, we investigated whether the potential export of heme from enterocytes by FLVCR1 present at the basolateral membrane was correlated with decreased plasma levels of Hpx. Using Western blotting we compared the levels of Hpx in the plasma of 3-day- and 14-day-old hemoglobin-supplemented piglets: the former group of animals had just started to receive supplementation, whereas the latter had been exposed to dietary hemoglobin for 11 days. This analysis showed a strong, age-dependent decline in plasma Hpx levels (Fig 6D). In contrast, plasma Hpx levels were unchanged in 3- and 14-day-old and anemic FeDex-supplemented piglets.

## Discussion

Dietary heme uptake by enterocytes has been recognized for more than 60 years [33]. Many studies over these decades have since confirmed that absorption of heme is far more efficient than that of inorganic iron [13,34]. However, our understanding of the molecular mechanisms of heme iron absorption remains poor. Recent mammalian studies have demonstrated several proteins involved in the transport of intact heme molecules at both the cellular and systemic levels [5–7,35]. Absorption of intact heme molecules by enterocytes might also contribute to systemic heme turnover under physiological conditions. Indeed, the recent discovery of a heme transporter that may transfer heme from the duodenum lumen directly into the enterocytes [17] and from enterocytes into the circulation [16], suggests a new putative pathway for trafficking intact heme across the enterocyte. Despite our limited knowledge of the molecular mechanisms of dietary absorption, heme preparations are successfully used to prevent and cure iron deficiency anemia in humans [12,14,31,36], dogs [37], and pigs [38].

In the present study we have examined the use of bovine hemoglobin, as a dietary heme supplement, in preventing IDA in piglets and investigated the duodenal expression profile of genes involved in heme iron absorption. Newborn piglets were chosen for our study because: (i) IDA is a well characterized iron disorder in pig neonates [22]; (ii) iron metabolism regulation has been extensively studied in these animals [19,39,40]; (iii) the pig is a major biomedical mammalian model in various human studies [41] that we have used previously for testing various strategies of iron supplementation [19–21]. The efficacy of hemoglobin treatment has been shown against a background of two groups of animals with an opposite iron status: iron-deficient and iron-replete piglets, intramuscularly injected with FeDex [20].

Dietary hemoglobin supplementation successfully prevented the development of IDA, which was observed in non-supplemented piglets. However, it is noteworthy that values of RBC indices and plasma iron parameters in *per os* hemoglobin-supplemented piglets were lower than in animals injected with large amount of FeDex, suggesting that their iron status was not fully replenished. We hypothesize that this was due to the immaturity of molecular mechanisms of heme iron absorption and the limited intake of solid feed by piglets during the first 10 days after birth. Importantly, our results provide evidence that dietary supplementation with hemoglobin stimulates recovery of piglets from severe neonatal IDA without inducing an unfavorable increase in hepcidin expression. With the aim of enhancing the curative effect of dietary hemoglobin, we successfully used a split iron supplementation regime consisting of the injection of a small amount of FeDex on day 3 after birth plus oral supplementation with hemoglobin. However, to avoid possible interference of intramuscularly given iron-heme iron the evaluation of expression of all analyzed genes was performed on samples obtained from piglets exclusively supplemented per os with hemoglobin.

To assess the potential contribution of dietary inorganic iron to the improvement of iron status in hemoglobin-supplemented piglets, we examined the expression of DMT1, a critical apical transmembrane transporter of ferrous ions expressed on the surface of the brush border of duodenal enterocytes [42]. The negligible influence of inorganic iron in the feed was indicated by the very low DMT1 mRNA and protein levels, as well as a residual presence at the apical membrane of duodenal enterocytes. Most importantly, our results imply that in the duodenum of hemoglobin-supplemented piglets, heme transporter(s) at the apical membrane of absorptive enterocytes are likely to provide the main driving force for the uptake of dietary iron. The occurrence of heme-binding proteins at the brush border of duodenal enterocytes in the pig was first suggested in 1970s/1980s [23,24]. However, HCP1, a plasma membrane protein responsible for the transfer of dietary heme from the intestinal lumen into absorptive enterocytes was only identified in mouse duodenum in 2005 [17]. Although its role as a heme carrier has since been questioned [18], several studies still support its involvement in low-affinity heme uptake [43–45]. Our results also suggest that HCP1 is involved in the uptake of dietary heme in hemoglobin-supplemented piglets, namely the specific up-regulation of its expression and enhanced HCP1 immunostaining at the apical membrane of duodenal enterocytes. Interestingly, the intracellular distribution of HCP1 in intestinal cells seems to be influenced by non-heme iron, i.e. under iron loading conditions HCP1 is mainly localized within the cytoplasm, while under conditions of iron deficiency the protein is found in the apical membrane [17]. Consistent with this posttranslational regulation, highly iron-loaded absorptive enterocytes from FeDex-injected piglets showed a weak, mainly intracellular distribution of HCP1, whereas in relatively iron-poor enterocytes from animals fed a hemoglobin-enriched diet it was predominantly localized to the brush border of absorptive epithelial cells.

Recent studies on *Caenorhabditis elegans*, a heme auxotroph, led to the identification of heme responsive gene 1 (HRG1), which has been proposed as a *bona fide* heme importer [30]. Although mammalian HRG1 shows an ubiquitous tissue distribution [30], its function and subcellular localization has been mostly studied in macrophages [15,46]. Immunolocalization and functional studies have revealed that mammalian HRG1 localizes to both the endolysosomal and the phagolysosomal compartments, and is involved in heme transport to the cytosol [15,25,46]. Two pieces of evidence led us to examine the involvement of HRG1 in heme absorption in piglets: the reported high level expression of the HRG1 gene in the mammalian small intestine and in cell lines derived from duodenum; the finding that *C*.*elegans* acquires dietary heme *via* an intestinal process involving HRG1 genes [30]. Western blot analysis of duodenal scraping extracts from piglets fed a hemoglobin-enriched diet showed a high level of HRG1 protein compared to control animals. Importantly, the intracellular localization of HRG1 in the apical membrane of enterocytes of hemoglobin-supplemented piglets was quite different from the dispersed pattern observed in enterocytes of FeDex-supplemented animals. Accordingly, HRG1 has been reported to be partially localized on the plasma membrane of many cell types [25,30,47]. This finding strongly suggests that HRG1 is involved in heme transport across the apical membrane. Studies on macrophages exposed to iron and heme revealed increased HRG1 expression [34,46]. It is therefore tempting to speculate that HRG1 expression, induced by increasing amounts of heme entering enterocytes and by heme-derived iron, occurs in enterocytes of piglets fed a hemoglobin-enriched diet.

The fate of heme upon entering the epithelial cells of the duodenum is mainly determined by the activity of HO1 [48], an enzyme that catalyzes the degradation of heme, releasing ferrous iron [49]. The proximal duodenum, the site of maximal heme absorption is also the site of the highest HO1 expression in the intestinal tract [48,50]. In most mammalian species, with the exception of mice [51], HO1-mediated heme catabolism in absorptive enterocytes seems to be the limiting step in dietary heme iron assimilation. Consequently, HO1 inhibitors have been shown to inhibit the absorption of heme iron [52]. We observed a profound induction of the *Hmox1* gene in the enterocytes of piglets fed a hemoglobin-enriched diet. IF analysis revealed high levels of HO1 distributed throughout the cytoplasm of their duodenal enterocytes. This specific and massive increase in HO1 in the duodenum of this group of animals strongly suggests that heme, released from hemoglobin by proteolytic activity in the lumen of the intestinal tract and transferred across the apical membrane as an intact molecule, is responsible for the up-regulation of the *Hmox1* gene. Indeed, apart from being a substrate of the enzymatic reaction catalyzed by HO1, heme has been reported to be a potent transcriptional inducer of *Hmox1* gene expression in various cell types [49] including enterocytes [48]. Activation of HO1 is closely correlated with the increase in the intracellular concentration of ferrous iron extracted from heme molecule [2]. This iron can be incorporated into ferritin molecules [53] and/or transported to the extracellular environment by ferroportin [32]. Accordingly, in duodenal enterocytes of piglets fed a hemoglobin-enriched diet we observed a concerted regulation of iron metabolism leading to the elevation of non-heme iron, an increased ferritin level, and up-regulation of Fpn expression. Fpn was distributed along the basolateral membrane of villus enterocytes, which is consistent with its typical localization in enterocytes of various mammalian species [54,55]. The extensive Fpn staining on the basolateral membrane of enterocytes indicates that under conditions producing a low plasma hepcidin-25 concentration, its binding to Fpn and the subsequent degradation of this iron exporter are reduced.This reflects the high potential of absorptive enterocytes to transfer iron released from heme to the bloodstream. The final part of our study examined the possibility that a portion of absorbed heme may be exported intact across the basolateral membrane of duodenal absorptive enterocytes into the bloodstream *via* a known heme export protein such as FLVCR1 [10]. In contrast to the well-defined role of FLVCR1 in the protection of several cell types [10,11,16,56] from potential heme toxicity, its function in duodenal heme metabolism has not been described. Nevertheless, it is noteworthy that the small intestine is one of the major sites of FLVCR1 expression in the body [11], which implies a potential role in heme absorption. Accordingly, postnatal mice lacking the *Slc49a1* gene show iron overload in duodenal enterocytes [11], indicating an active role for FLVCR1 in transferring intact heme to the circulation. Here, we showed that FLVCR1 is highly expressed in hemoglobin-supplemented piglets, mainly at the basolateral membrane of duodenal enterocytes, suggesting its involvement in the transport of heme taken up from the diet (but not catabolized by HO1) into the bloodstream. Further supposition regarding such a mechanism operating in the enterocytes of piglets fed a hemoglobin-enriched diet is based on our observations of the behavior of Hpx in the plasma of these animals during the initial period of exposure to dietary hemoglobin. It was previously shown that heme export via FLVCR1 requires the presence of the extracellular heme-binding protein Hpx [25]. Furthermore, Hpx-null mice displayed iron loading in the duodenum [57]. Serum levels of Hpx are usually low under conditions of increased heme flux to the circulation, because Hpx binds free heme and then the Hpx-heme complex is rapidly removed by the scavenger receptor CD91 [9,58]. Consistent with this scenario, the strong decrease in plasma Hpx levels detected in 14-day-old piglets fed hemoglobin suggests that in these animals, Hpx actively binds heme exported from enterocytes *via* FLVCR1 and then participates in its distribution throughout the body.

**Fig 7:**
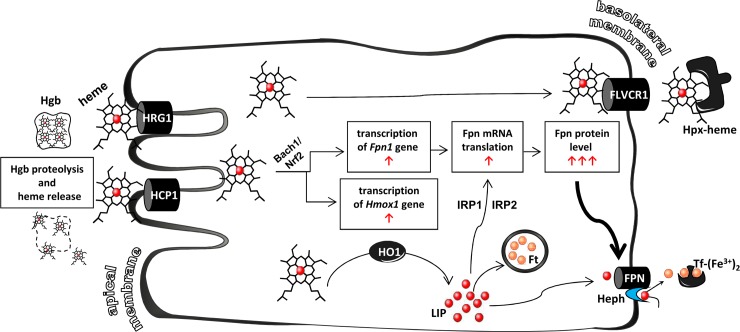
Proposed pathways and regulatory mechanisms of heme iron absorption in piglets supplemented with dietary hemoglobin. Before heme iron can be utilized, heme must be extracted from hemoglobin by proteolytic activity in the stomach and duodenum. Intestinal heme uptake occurs in the proximal part of the duodenum. The first step in heme iron absorption involves intact heme transport across the apical membrane to the enterocyte interior *via* the HCP1 and HRG1 importers. The passage of heme iron through the enterocyte then divides into two separate pathways. First, intracytoplasmic heme can be catabolized by heme oxygenase 1 (HO1), a process leading to the release of ferrous iron. Freed heme iron is then neutralized by ferritin, a cytosolic multimeric iron-storage protein, or recycled to the circulation *via* ferroportin (Fpn), where it is bound by transferrin (Tf). Free heme, apart from being a substrate of the enzymatic reaction catalyzed by HO1, may induce the expression of both *Hmox1* and *Fpn* genes at the transcriptional level *via* the MARE/ARE sequence motif, a regulatory mechanism that involves inactivation of the transcriptional repressor Bach1 and the recruitment of the transcriptional activator Nrf2. In addition, iron extracted from the protoporphyrin ring of heme can promote the translation of the Fpn transcript containing the Iron Responsive Element (IRE) in its 5’ UTR *via* the Iron Regulatory Proteins. Finally, up-regulation of Fpn on the enterocyte basolateral surface of hemoglobin-fed piglets may be due to the reduced binding of hepcidin (showing a residual concentration in the blood plasma) to cell surface Fpn and thus to its limited internalization and degradation. Orchestrated up-regulation of Fpn expression in duodenal enterocytes at different regulatory steps may be a key event in understanding high heme bioavailability. The second, minor, pathway of heme iron movement involves the transport of intact heme, not catabolized by HO1, across the basolateral membrane by FLVCR1 to the plasma, where it is captured by hemopexin (Hpx) and delivered in the form of heme-hemopexin via receptor CD91 to various sites in the body.

Taken together, the results of this study demonstrate that dietary hemoglobin can efficiently rescue piglets from severe iron deficiency anemia. Importantly, our results clearly show that giving heme as an oral iron supplement to iron-deficient subjects may overcome the main disadvantage associated with oral supplementation based on elemental iron, which consists on the induction of hepcidin expression and subsequent inhibition of iron absorption from the diet [59]. Considering that the pig is a major biomedical mammalian model for human studies, our findings should help to improve existing protocols of IDA correction. Our data also provide the framework for a long sought after comprehensive molecular model of heme iron absorption, summarized in Fig 7.

## Materials and methods

### Piglets, experimental design and biological sample collection

Experiments were conducted at the Brzezie pig farm belonging to the National Research Institute of Animal Production (NRIAP, Balice, Poland). A total of 39 Polish Landrace x Polish Large White piglets housed in standard conditions (70% humidity and a temperature of 22°C in cages with straw bedding) were used in experiments. During the 28-day experiment sows were allowed to nurse their piglets. Importantly, piglets had no access to the sows’ feed. The Prestarter Wigor 1 Plus feed (manufactured at the feed mill of the Experimental Station of the NRIAP) was offered to piglets from day 3 to day 28 after birth using specially designed feeding troughs (Play-feeder; Biofiber-Damino), the height of which was adjusted to the age and size of the piglets [60]. Piglets were allotted to 3 experimental groups on the basis of balanced body weight (b.w.) at birth: (i) piglets receiving no iron supplementation, n = 15; (ii) piglets supplemented parenterally with 150 and 40 mg Fe/kg b.w. on days 3 and 21 postpartum, respectively (routine supplementation), n = 9. Piglets received intramuscular injections in the neck of iron dextran (FeDex), a complex of ferric ions with low molecular weight dextran (Ferran 100, Vet-Agro, Lublin, Poland); (iii) Piglets fed Prestarter Wigor 1 Plus feed enriched with bovine hemoglobin (Bovogen, East Keilor, Australia) from day 3 to day 28 after birth, n = 15. Bovine hemoglobin was added to the feed in the proportion 38 g hemoglobin *per* 1 kg of feed. The final total iron content in this mixture, assessed by flame atomic absorption spectroscopy as described previously [61], was 612 mg Fe/kg. The mean daily per piglet consumption of feed and the respective calculated iron intake were monitored in the 3 experimental groups, and are shown in S1 Table. The use of animals in experiments and all procedures were approved by the Third Local Ethical Committee on Animal Testing (permission no. 55/2012).

Blood was drawn on days 3, 14, 21 and 28 after birth by venipuncture of the jugular vein (*Vena jugularis externa*) into tubes coated with heparin as an anticoagulant. The blood samples were immediately centrifuged (2000 rpm, 10 min, 4°C) to separate the plasma. Plasma samples were immediately aliquoted and stored at -80°C. 28-day-old piglets were euthanized by the intracardiac injection of Morbital (Biowet, Puławy, Poland).

The proximal segment (5 cm) of the duodenum downstream of the stomach was dissected *post mortem* from piglets, carefully washed with PBS and cut into two equal parts. One part was used for immunostaining and iron staining analyses. The other part was further dissected to obtain a highly enriched epithelium fraction. A lancet was used to scrape the upper layer of the duodenum, making efforts to avoid the circular muscle. Duodenal scrapings were then stored at -80°C until they were used for Western blotting and RT-qPCR analyses.

### Measurement of red blood cell indices and plasma iron concentration

The red blood cell (RBC) count, hemoglobin (HGB) concentration and mean hematocrit (HCT) value were determined using an automated ADVIA 2010 analyzer (Siemens. Germany). The plasma iron concentration was determined by colorimetric measurement of an iron-chromazurol complex (absorbance at 630 nm) according to the manufacturer’s protocol (Alpha Diagnostic, Poland).

### Protein extract preparation and Western blotting

For the analysis of most proteins crude membrane and cytosolic protein extracts were prepared from duodenal scrapings as described previously [27,28]. For the detection of HRG1, frozen duodenal scrapings samples were suspended in membrane preparation buffer (10mM Tris-HCl, pH 7.4, 250mM sucrose, 1mM EDTA, proteases inhibitors), homogenized, centrifuged (800x*g*, 10 minutes, 4°C) and supernatants were ultracentrifuged at 100,000x*g* for 2 hours at 4°C. After removing supernatant, 40–100μl of lysis buffer (20mM Hepes pH 7.4, 150mM NaCl, 1mM EDTA, 2% Triton-X, proteases inhibitors) was added to the membrane fraction pellet and resuspended by vortexing. Samples were centrifuged at 18,000 x *g* for 15 minutes, 4°C to pellet insoluble fraction. Supernatant was separated for protein concentration analysis. For all Western blot analyzes, Laemmli sample buffer was added to samples and samples were performed according to the Laemmli SDS PAGE procedure. A list of primary and secondary antibodies used is shown in S3 Table. All antibodies used in the study show similar reactivity with mouse and pig proteins as shown in S1 Fig. Additionally we performed Blastn and Blastp analysis for analyzed genes and proteins and results are shown in S5 Table.

### Real-time quantitative PCR (RT-qPCR)

Total cellular RNA was extracted from duodenal scrapings (20 mg) using Trizol reagent (Invitrogen) according to the manufacturer’s protocol. Two micrograms of total DNAse-treated RNA were reverse transcribed using a Transcriptor First Strand cDNA Synthesis Kit^®^ (Roche, Switzerland). Real-time quantitative PCR analysis was performed in a Light Cycler U96 (Roche Diagnostics, Mannheim, Germany) using gene-specific primer pairs (S4 Table). The amplified products were detected using SYBR Green I (Roche Diagnostics) as described previously [62]. To confirm amplification specificity, the PCR products were subjected to melting curve analysis and agarose gel electrophoresis. Light Cycler U96 Software was used for data analysis. Transcript levels were normalized relative to those of the *18S* rRNA and *glutathione reductase* (GSR) control reference genes selected using NormFinder software (http://www.mdl.dk/publicationsnormfinder.htm).

### Immunofluorescence (IF) and confocal microscopy analysis of duodenal sections

After the sacrifice of piglets, samples of proximal duodenum were immediately excised and fixed in 4% paraformaldehyde (Sigma-Aldrich) in phosphate-buffered saline (PBS) (Sigma) at 4°C for 24 h. After washing 3 times for 30 minutes in PBS, the fixed samples were successively soaked in 12.5 and 25% sucrose (Merck) for 1.5 and 12 hours, respectively at 4°C. The tissue was then embedded in Tissue-Tek compound, frozen in liquid nitrogen and sectioned into 20-μm slices using a cryostat (Leica). The sections were washed in PBS and permeabilized by bathing in PBS/0.1% Triton X-100 (Sigma) for 10 minutes. Non-specific antibody binding was blocked by incubation of the sections in PBS/3% BSA (Merck) for 1.5 hours. For protein detection, sections were incubated at RT with primary antibody (S3 Table) diluted in PBS/3% BSA. The sections were then washed 3 times with PBS and incubated with Cy3 (indocarbocyanine)-conjugated secondary antibody (S3 Table) diluted in PBS/3% BSA. Finally, the sections were washed 3 times for 10 minutes in PBS at RT and mounted using Vectashield with 4′,6-diamidine-2-phenylindole (DAPI; Vector Labs). As a negative control, some sections were prepared without incubating with primary antibody. IF was analyzed with a Zeiss LSM 510 Meta confocal microscope (Carl Zeiss, Jena, Germany) using the 60x objective.

### Duodenal iron staining

Non-heme iron deposits were analyzed using the Accustain Iron Deposition Kit (Sigma Aldrich). Briefly, duodenal samples excised immediately after sacrifice were fixed in Bouin’s solution for 24 h, then stored in 70% ethanol. After embedding in paraffin, the samples were cut into 7-μm sections using a microtome (Reichert-Jung, Germany). The sections were placed on a slide, deparaffinized and incubated with Perls’ Prussian Blue solution for 30 minutes. Slides were counterstained with pararoseaniline solution for 2 minutes and examined with a standard compound light microscope (Olympus, type CH2).

### Plasma hepcidin-25 quantification

Piglet plasma hepcidin-25 measurements were performed using a combination of weak cation exchange chromatography and time-of-flight mass spectrometry (WCX-TOF MS), as described previously for human and porcine plasma samples [21,63]. Piglet plasma hepcidin-25 concentrations were expressed as nmol/L (nM).

### Statistical analysis

Data are presented as mean values ± standard deviation (SD). Statistical analysis was performed using GraphPad Prism software (GraphPad, San Diego, CA, USA) by one-way analysis of variance (ANOVA), followed by Tukey-Kramer *post hoc* test. p≤0.05, p≤0.01, and p≤0.001 were considered significant and are denoted with one, two or three asterisks, respectively.

## Supporting information

S1 Fig Validation of the cross-reactivity of antibodies with pig proteins analyzed in the study. Comparative Western blot analysis shows that primary antibodies cross-react with mouse as well as with pig proteins. For the analysis crude membrane and cytosolic protein extracts (40–60 μg protein) were prepared from duodenal scrapings as described previously in Material and methods. Immunolabelled protein bands from blots performed on scrapings isolated from 4 mice and 4 piglets are shown.
(TIF)

S1 Table Mean daily feed and iron intake by piglets during 4 main periods after birth. (DOCX)

S2 Table Mean body weight gain in piglets from the 1^st^ to the 28^th^ day after birth (mean ± S.D.). (DOCX)

S3 Table List of antibodies used for Western blot analyses. (DOCX)

S4 Table List of oligonucleotide primers used for RT-qPCR. (DOCX)

S5 Table Blastn and blastp analysis for examined genes and proteins. (DOCX)

## References

[1] Lin YW, Wang J: Structure and function of heme proteins in non-native states: A mini-review. J Inorg Biochem 2013, 129:162–171 doi: 10.1016/j.jinorgbio.2013.07.023 23916118 10.1016/j.jinorgbio.2013.07.023

[2] Kikuchi G, Yoshida T, Noguchi M: Heme oxygenase and heme degradation. Biochem Biophys Res Commun 2005, 338:558–567 doi: 10.1016/j.bbrc.2005.08.020 16115609 10.1016/j.bbrc.2005.08.020

[3] Kühn LC: Iron regulatory proteins and their role in controlling iron metabolism. Metallomics 2015, 7:232–43 doi: 10.1039/c4mt00164h 25306858 10.1039/c4mt00164h

[4] Ganz T, Nemeth E: Hepcidin and iron homeostasis. Biochim Biophys Acta 2012, 1823:1434–43 doi: 10.1016/j.bbamcr.2012.01.014 22306005 10.1016/j.bbamcr.2012.01.014 PMC4048856

[5] Chiabrando D, Vinchi F, Fiorito V, Mercurio S, Tolosano E: Heme in pathophysiology: a matter of scavenging, metabolism and trafficking across cell membranes. Front Pharmacol 2014, 5:61 doi: 10.3389/fphar.2014.00061 24782769 10.3389/fphar.2014.00061 PMC3986552

[6] Khan AA, Quigley JG: Control of intracellular heme levels: heme transporters and heme oxygenases. Biochim Biophys Acta 2011, 1813:668–682 doi: 10.1016/j.bbamcr.2011.01.008 21238504 10.1016/j.bbamcr.2011.01.008 PMC3079059

[7] Khan AA, Quigley JG: Heme and FLVCR-related transporter families SLC48 and SLC49. Mol Aspects Med 2013, 34:669–682 doi: 10.1016/j.mam.2012.07.013 23506900 10.1016/j.mam.2012.07.013 PMC3602793

[8] Severance S, Hamza I: Trafficking of heme and porphyrins in metazoa. Chem Rev 2009, 109:4596–616 doi: 10.1021/cr9001116 19764719 10.1021/cr9001116 PMC2769250

[9] Hvidberg V, Maniecki MB, Jacobsen C, Højrup P, Møller HJ, Moestrup SK: Identification of the receptor scavenging hemopexin-heme complexes. Blood 2005, 106:2572–2579 doi: 10.1182/blood-2005-03-1185 15947085 10.1182/blood-2005-03-1185

[10] Vinchi F, Ingoglia G, Chiabrando D, Mercurio S, Turco E, Silengo L, et al: Heme exporter FLVCR1a regulates heme synthesis and degradation and controls activity of cytochromes P450. Gastroenterology 2014, 146:1325–38. doi: 10.1053/j.gastro.2014.01.053 24486949 10.1053/j.gastro.2014.01.053 PMC4000440

[11] Keel SB, Doty RT, Yang Z, Quigley JG, Chen J, Knoblaugh S, et al: A heme export protein is required for red blood cell differentiation and iron homeostasis. Science 2008, 319:825–828 doi: 10.1126/science.1151133 18258918 10.1126/science.1151133

[12] Young MF, Griffin I, Pressman E, McIntyre AW, Cooper E, McNanley T, Harris ZL, Westerman M, O’Brien KO: Utilization of iron from an animal-based iron source is greater than that of ferrous sulfate in pregnant and nonpregnant women. J: Nutr 2010, 140:2162–2166 20980658 10.3945/jn.110.127209 PMC2981003

[13] Anderson GJ, Frazer DM, McKie AT, Vulpe CD, Smith A: Mechanisms of haem and non-haem iron absorption: lessons from inherited disorders of iron metabolism. Biometals 2005, 18:339–48 doi: 10.1007/s10534-005-3708-8 16158226 10.1007/s10534-005-3708-8

[14] Walsh RJ, Kaldor I, Brading I, George EP: The availability of iron in meat: some experiments with radioactive iron. Australas Ann Med 1955, 4:272–6 13293105 10.1111/imj.1955.4.4.272

[15] Yanatori I, Tabuchi M, Kawai Y, Yasui Y, Akagi R, Kishi F: Heme and non-heme iron transporters in non-polarized and polarized cells. BMC Cell Biol 2010, 11:39 doi: 10.1186/1471-2121-11-39 20525315 10.1186/1471-2121-11-39 PMC3224662

[16] Fiorito V, Forni M, Silengo L, Altruda F, Tolosano E: Crucial Role of FLVCR1a in the Maintenance of Intestinal Heme Homeostasis. Antioxid Redox Signal 2015, 23:1410–23 doi: 10.1089/ars.2014.6216 26067085 10.1089/ars.2014.6216

[17] Shayeghi M, Latunde-Dada GO, Oakhill JS, Laftah AH, Takeuchi K, Halliday N, et al: Identification of an intestinal heme transporter. Cell 2005, 122:789–801 doi: 10.1016/j.cell.2005.06.025 16143108 10.1016/j.cell.2005.06.025

[18] Qiu A, Jansen M, Sakaris A, Min SH, Chattopadhyay S, Tsai E, et al: Identification of an intestinal folate transporter and the molecular basis for hereditary folate malabsorption. Cell 2006, 127:917–28 doi: 10.1016/j.cell.2006.09.041 17129779 10.1016/j.cell.2006.09.041

[19] Lipiński P, Starzyński RR, Canonne-Hergaux F, Tudek B, Oliński R, Kowalczyk P, et al: Benefits and risks of iron supplementation in anemic neonatal pigs. Am J Pathol 2010, 177:1233–43 doi: 10.2353/ajpath.2010.091020 20805566 10.2353/ajpath.2010.091020 PMC2928957

[20] Staroń R, Van Swelm RP, Lipiński P, Gajowiak A, Lenartowicz M, Bednarz A, et al: Urinary Hepcidin Levels in Iron-Deficient and Iron-Supplemented Piglets Correlate with Hepcidin Hepatic mRNA and Serum Levels and with Body Iron Status. PLoS One 2015, 10:e0136695 doi: 10.1371/journal.pone.0136695 26323096 10.1371/journal.pone.0136695 PMC4556373

[21] Starzyński RR, Laarakkers CM, Tjalsma H, Swinkels DW, Pieszka M, Styś A, et al: Iron supplementation in suckling piglets: how to correct iron deficiency anemia without affecting plasma hepcidin levels. PLoS One 2013, 8:e64022 doi: 10.1371/journal.pone.0064022 23737963 10.1371/journal.pone.0064022 PMC3667775

[22] Svoboda M, Drabek J: Iron deficiency in suckling piglets: etiology, clinical aspects and diagnosis. Folia Vet 2005, 49:104–111

[23] Gräsbeck R, Kouvonen I, Lundberg M, Tenhunen R: An intestinal receptor for heme. Scand J Haematol 1979, 23:5–9 493872 10.1111/j.1600-0609.1979.tb02845.x

[24] Gräsbeck R, Majuri R, Kouvonen I, Tenhunen R: Spectral and other studies on the intestinal haem receptor of the pig. Biochim Biophys Acta 1982, 700: 137–42 6275896 10.1016/0167-4838(82)90089-9

[25] Yang Z, Philips JD, Doty RT, Giraudi P, Ostrow JD, Tiribelli C, et al: Kinetics and specificity of feline leukemia virus subgroup C receptor (FLVCR) export function and its dependence on hemopexin. J Biol Chem 2010, 285:28874–28882 doi: 10.1074/jbc.M110.119131 20610401 10.1074/jbc.M110.119131 PMC2937914

[26] Egeli AK, Framstad T, Morberg H. Clinical biochemistry, haematology and body weight in piglets: Acta Vet Scand 1998, 39:381–93 9787501 10.1186/BF03547786 PMC8050675

[27] Canonne-Hergaux F, Gruenheid S, Ponka P, Gros P: Cellular and subcellular localization of the Nramp2 iron transporter in the intestinal brush border and regulation by dietary iron. Blood 1999, 93:4406–17 10361139

[28] Canonne-Hergaux F, Fleming MD, Levy JE, Gauthier S, Ralph T, Picard V, et al: The Nramp2/DMT1 iron transporter is induced in the duodenum of microcytic anemia mk mice but is not properly targeted to the intestinal brush border. Blood 2000, 96:3964–70 11090085

[29] Galy B, Ferring-Appel D, Kaden S, Gröne HJ, Hentze MW. Iron regulatory proteins are essential for intestinal function and control key iron absorption molecules in the duodenum. Cell Metab. 2008 7:79–85. doi: 10.1016/j.cmet.2007.10.006 18177727 10.1016/j.cmet.2007.10.006

[30] Rajagopal A, Rao AU, Amigo J, Tian M, Upadhyay SK, Hall C, et al: Haem homeostasis is regulated by the conserved and concerted functions of HRG-1 proteins. Nature 2008, 453:1127–31 doi: 10.1038/nature06934 18418376 10.1038/nature06934 PMC4058867

[31] Gonzalez-Rosendo G, Polo J, Rodrıguez-Jerez JJ, Puga-Dıaz R, Reyes-Navarrete EG, Quintero-Gutierrez AG: Bioavailability of a heme-iron concentrate product added to chocolate biscuit filling in adolescent girls living in a rural area of Mexico. J Food Sci 2010, 75:H73–H78 doi: 10.1111/j.1750-3841.2010.01523.x 20492296 10.1111/j.1750-3841.2010.01523.x

[32] Donovan A, Lima CA, Pinkus JL, Pinkus GS, Zon LI, Robine S, et al: The iron exporter ferroportin/Slc40a1 is essential for iron homeostasis. Cell Metab 2005, 1:191–200 doi: 10.1016/j.cmet.2005.01.003 16054062 10.1016/j.cmet.2005.01.003

[33] West AR, Oates PS: Subcellular location of heme oxygenase 1 and 2 and divalent metal transporter 1 in relation to endocytotic markers during heme iron absorption. J Gastroenterol Hepatol 2008, 23:150–8 doi: 10.1111/j.1440-1746.2007.05047.x 17614955 10.1111/j.1440-1746.2007.05047.x

[34] White C, Yuan X, Schmidt PJ, Bresciani E, Samuel TK, Campagna D, et al: HRG1 is essential for heme transport from the phagolysosome of macrophages during erythrophagocytosis. Cell Metab 2013, 17:261–70 doi: 10.1016/j.cmet.2013.01.005 23395172 10.1016/j.cmet.2013.01.005 PMC3582031

[35] Ogawa K, Sun J, Taketani S, Nakajima O, Nishitani C, Sassa S, et al: Heme mediates derepression of Maf recognition element through direct Winding to transcription repressor Bach1. EMBO J 2001, 20:2835–2843 doi: 10.1093/emboj/20.11.2835 11387216 10.1093/emboj/20.11.2835 PMC125477

[36] Young MF, Griffin I, Pressman E, McIntyre AW, Cooper E, McNanley T, et al: Maternal hepcidin is associated with placental transfer of iron derived from dietary heme and nonheme sources. J Nutr 2012, 142:33–9 doi: 10.3945/jn.111.145961 22113871 10.3945/jn.111.145961 PMC3237230

[37] Weintraub LR, Weinstein MB, Huser HJ, Rafal S. Absorption of hemoglobin iron: the role of a heme-splitting substance in the intestinal mucosa. J Clin Invest. 1968; 47(3):531–9. doi: 10.1172/JCI105749 5637141 10.1172/JCI105749 PMC297199

[38] Quintero-Gutierrez AG, Gonzalez-Rosendo G, Sanchez-Munoz J, Polo-Pozo J, Rodrıguez-Jerez JJ: Bioavailability of heme iron in biscuit filling using piglets as an animal model for humans. Int J Biol Sci 2008, 4:58–62 18311330 10.7150/ijbs.4.58 PMC2253952

[39] Blachier F, Vaugelade P, Robert V, Kibangou B, Canonne-Hergaux F, Delpal S, et al: Comparative capacities of the pig colon and duodenum for luminal iron absorption. Can J Physiol Pharmacol 2007, 85:85–92 10.1139/y07-007 17487259

[40] Rincker MJ, Clarke SL, Eisenstein RS, Link JE, Hill GM: Effects of iron supplementation on binding activity of iron regulatory proteins and the subsequent effect on growth performance and indices of hematological and mineral status of young pigs. J Anim Sci 2005, 83:2137–2145 16100069 10.2527/2005.8392137x

[41] Schook L, Beattie C, Beever J, Donovan S, Jamison R, Zuckermann F, et al: Swine in biomedical research: creating the building blocks of animal models. Anim Biotechnol 2005, 16:183–190 16342425 10.1080/10495390500265034

[42] Andrews NC: The iron transporter DMT1. Int J Biochem Cell Biol 1999, 31:991–4 10582331 10.1016/s1357-2725(99)00065-5

[43] Laftah AH, Latunde-Dada GO, Fakih S, Hider RC, Simpson RJ, McKie AT: Haem and folate transport by proton-coupled folate transporter/haem carrier protein 1 (SLC46A1). Br J Nutr 2009, 101:1150–6: doi: 10.1017/S0007114508066762 18782461 10.1017/S0007114508066762

[44] Latunde-Dada GO, Takeuchi K, Simpson RJ, McKie AT: Haem carrier protein 1 (HCP1): Expression and functional studies in cultured cells. FEBS Lett 2006, 580:6865–70 doi: 10.1016/j.febslet.2006.11.048 17156779 10.1016/j.febslet.2006.11.048

[45] Le Blanc S, Garrick MD, Arredondo M: Heme carrier protein 1 transports heme and is involved in heme-Fe metabolism. Am J Physiol Cell Physiol 2012, 302:C1780–5 doi: 10.1152/ajpcell.00080.2012 22496243 10.1152/ajpcell.00080.2012

[46] Delaby C, Rondeau C, Pouzet C, Willemetz A, Pilard N, Desjardins M, et al: Subcellular localization of iron and heme metabolism related proteins at early stages of erythrophagocytosis. PLoS One 2012, 7:e42199 doi: 10.1371/journal.pone.0042199 22860081 10.1371/journal.pone.0042199 PMC3408460

[47] O’Callaghan KM, Ayllon V, O’Keeffe J, Wang Y, Cox OT, Loughran G, et al: Heme-binding protein HRG-1 is induced by insulin-like growth factor I andassociates with the vacuolar H+-ATPase to control endosomal pH and receptor trafficking. J Biol Chem 2010, 285:381–391 doi: 10.1074/jbc.M109.063248 19875448 10.1074/jbc.M109.063248 PMC2805445

[48] West AR, Oates PS: Mechanisms of heme iron absorption: Current questions and controversies. World J Gastroenterol 2008, 14926:4101–4110 10.3748/wjg.14.4101 PMC2725368 18636652

[49] Ponka P: Cell biology of heme. Am J Med: Sci 1999, 318:241–256 10522552 10.1097/00000441-199910000-00004

[50] Collins JF, Franck CA, Kowdley KV, Ghishan FK: Identification of differentially expressed genes in response to dietary iron deprivation in rat duodenum. Am J Physiol Gastrointest Liver Physiol 2005, 288:G964–71 doi: 10.1152/ajpgi.00489.2004 15637178 10.1152/ajpgi.00489.2004

[51] Fillebeen C, Gkouvatsos K, Fragoso G, Calvé A, Garcia-Santos D, Buffler M, et al: Mice are poor heme absorbers and do not require intestinal Hmox1 for dietary heme iron assimilation. Haematologica 2015,100:e334–7 doi: 10.3324/haematol.2015.126870 25975840 10.3324/haematol.2015.126870 PMC4800685

[52] Böni RE, Huch Böni RA, Galbraith RA, Drummond GS, Kappas A: Tin-mesoporphyrin inhibits heme oxygenase activity and heme-iron absorption in the intestine. Pharmacology 1993, 47:318–29 8265722 10.1159/000139113

[53] Gozzelino R, Soares MP: Coupling heme and iron metabolism via ferritin H chain. Antioxid Redox Signal 2014, 20:1754–69 doi: 10.1089/ars.2013.5666 24124891 10.1089/ars.2013.5666 PMC3961798

[54] Canonne-Hergaux F, Donovan A, Delaby C, Wang HJ, Gros P: Comparative studies of duodenal and macrophage ferroportin proteins. Am J Physiol Gastrointest Liver Physiol 2006, 290:G156–63 doi: 10.1152/ajpgi.00227.2005 16081760 10.1152/ajpgi.00227.2005

[55] Drakesmith H, Nemeth E, Ganz T: Ironing out ferroportin. Cell Metab 2015, 22:777–87 doi: 10.1016/j.cmet.2015.09.006 26437604 10.1016/j.cmet.2015.09.006 PMC4635047

[56] Vinchi F, De Franceschi L, Ghigo A, Townes T, Cimino J, Silengo L, et al: Hemopexin therapy improves cardiovascular function by preventing heme-induced endothelial toxicity in mouse models of hemolytic diseases. Circulation 2013, 127:1317–29 doi: 10.1161/CIRCULATIONAHA.112.130179 23446829 10.1161/CIRCULATIONAHA.112.130179

[57] Fiorito V, Geninatti Crich S, Silengo L, Aime S, Altruda F, Tolosano E: Lack of Plasma Protein Hemopexin Results in Increased Duodenal Iron Uptake. PLoS One 2013, 8:e68146 doi: 10.1371/journal.pone.0068146 23826373 10.1371/journal.pone.0068146 PMC3694894

[58] Tolosano E, Fagoonee S, Morello N, Vinchi F, Fiorito V: Heme scavenging and the other facets of hemopexin. Antioxid Redox Signal 2010, 12:305–320 doi: 10.1089/ars.2009.2787 19650691 10.1089/ars.2009.2787

[59] Moretti D, Goede JS, Zeder C, Jiskra M, Chatzinakou V, Tjalsma H, et al: Oral iron supplements increase hepcidin and decrease iron absorption from daily or twice-daily doses in iron-depleted young women. Blood 2015, 126:1981–9 doi: 10.1182/blood-2015-05-642223 26289639 10.1182/blood-2015-05-642223

[60] Maes D, Steyaert M, Vanderhaeghe C, López Rodríguez A, de Jong E, et al: Comparison of oral versus parenteral iron supplementation on the health and productivity of piglets. Vet Rec 2011, 168:188 doi: 10.1136/vr.c7033 21493531 10.1136/vr.c7033

[61] Starzyński RR, Lipiński P, Drapier JC, Diet A, Smuda E, Bartlomiejczyk T, et al: Down-regulation of iron regulatory protein 1 activities and expression in superoxide dismutase 1 knock-out mice is not associated with alterations in iron metabolism. J Biol Chem 2005, 280:4207–12 doi: 10.1074/jbc.M411055200 15557328 10.1074/jbc.M411055200

[62] Starzyński RR, Canonne-Hergaux F, Lenartowicz M, Krzeptowski W, Willemetz A, Styś A, et al: Ferroportin expression in haem oxygenase 1-deficient mice. Biochem J 2013a, 449:69–78 22992020 10.1042/BJ20121139

[63] Kroot JJ, Laarakkers CM, Geurts-Moespot AJ, Grebenchtchikov N, Pickkers P, van Ede AE, et al: Immunochemical and mass-spectrometry-based serum hepcidin assays for iron metabolism disorders. Clin Chem 2010, 56:1570–9. doi: 10.1373/clinchem.2010.149187 20739637 10.1373/clinchem.2010.149187

