# Supplementary material for: Dietary hemoglobin rescues young piglets from severe iron deficiency anemia: Duodenal expression profile of genes involved in heme iron absorption
Source: PLoS One. 2017 Jul 13;12(7):e0181117. doi: 10.1371/journal.pone.0181117 (PMC5514692; doi:10.1371/journal.pone.0181117)
Supplement: S1 Table — (DOCX) [file pone.0181117.s002.docx]

**S1 Table.** Mean daily feed and iron intake by piglets during 4 main periods after birth

| **Age (Days)**    **Group** | **Period I: 3-9 day**  **(6.13% of total**  **feed intake)** | | **Period II: 10-16 day**  **(10.9% of total**  **feed intake)** | | **Period III: 17-23 day**  **(28.87% of total**  **feed intake)** | | **Period IV: 24-28 day**  **(54.1% of total**  **feed intake)** | |
| --- | --- | --- | --- | --- | --- | --- | --- | --- |
|  | **Feed**  **(g)** | **Fe**  **(mg)** | **Feed**  **(g)** | **Fe**  **(mg)** | **Feed**  **(g)** | **Fe**  **(mg)** | **Feed**  **(g)** | **Fe**  **(mg)** |
| **Control** | **1.81** | **0.43** | **3.23** | **0.77** | **8.55** | **2.03** | **16.01** | **3.81** |
| **Iron dextran** | **1.95** | **0.46** | **3.46** | **0.82** | **9.17** | **2.18** | **17.17** | **4.09** |
| **Hemoglobin** | **2.06** | **1.26** | **3.66** | **2.24** | **9.70** | **5.94** | **18.18** | **11.13** |
