# Supplementary material for: Dietary hemoglobin rescues young piglets from severe iron deficiency anemia: Duodenal expression profile of genes involved in heme iron absorption
Source: PLoS One. 2017 Jul 13;12(7):e0181117. doi: 10.1371/journal.pone.0181117 (PMC5514692; doi:10.1371/journal.pone.0181117)
Supplement: S2 Table — (DOCX) [file pone.0181117.s003.docx]

**S2 Table.** Mean body weight gain in piglets from the 1^st^ to the 28^th^ day after birth (mean ± S.D.)

| **Parameter**  **Group** | **Body weight gain (kg)** |
| --- | --- |
| **Control** | **5.75 ±** 0.88 |
| **Iron dextran** | **6.51 ±** 0.72 |
| **Hemoglobin** | **6.32 ±** 1.37 |
