# Supplementary material for: Dietary hemoglobin rescues young piglets from severe iron deficiency anemia: Duodenal expression profile of genes involved in heme iron absorption
Source: PLoS One. 2017 Jul 13;12(7):e0181117. doi: 10.1371/journal.pone.0181117 (PMC5514692; doi:10.1371/journal.pone.0181117)
Supplement: S4 Table — (DOCX) [file pone.0181117.s005.docx]

**S4 Table.** List of oligonucleotide primers used for RT-qPCR

| **Gene** | **Encoded product** | **Forward (5’-3’)** | **Reverse (5’-3’)** | **PCR product size [bp]** |
| --- | --- | --- | --- | --- |
| *18S* | 18S rRNA | AGGAAAGCAGACATCGACCT | ACCTGGCTGTACTTCCCATC | 158 |
| *Gsr* | Glutathione reductase | CACAGCTCCTCACATCCTGA | GGGCAATTCTTCCAGCTGAA | 121 |
| *Slc12a1* | Divalent metal transporter 1 | GCAGTCCCCATAGTGACCTT | AGGTTCCTGTCATGGTGGAG | 162 |
| *Hpx* | Hemopexin | TGGAATGTCACCGTGGAGAA | GTTACCCCGGAAGCAGTAGT | 173 |
| *Hmox1* | Heme oxygenase 1 | ATGGCGTCCTTGTACCACAT | AGACAGGTCACCCATGTAGC | 237 |
| *Slc40a1* | Ferroportin | TCGCCTAGTGTCATGACCAG | CAGAAACACAGACACCGCAA | 162 |
| *Hepc* | Hepcidin | AAGACAGCTCACAGACCTCC | CTACGTCTTGCAGCACATCC | 160 |
| *Slc46a1* | Heme carrier protein 1 | CTGGGACTCTAGGCTGATCG | AGTGATGACCAGCGACAGAA | 223 |
