# Supplementary material for: Dietary hemoglobin rescues young piglets from severe iron deficiency anemia: Duodenal expression profile of genes involved in heme iron absorption
Source: PLoS One. 2017 Jul 13;12(7):e0181117. doi: 10.1371/journal.pone.0181117 (PMC5514692; doi:10.1371/journal.pone.0181117)
Supplement: S5 Table — (DOCX) [file pone.0181117.s006.docx]

**S5 Table.** Blastn and blastp analysis for examined genes and proteins.

| **Target**  **protein** | **epitope** | **Primary Ab** | **Dilution** | **Epitop seq./sus scrofa cDNA and protein seq. alignments**  **(% of identity)**  **According to: GeneBank and Uniprot** | **Secondary Ab** | **Dilution** |
| --- | --- | --- | --- | --- | --- | --- |
| Hrg1 | human | Rabbit polyclonal, kind gift from Dr. I. Hamza, University of Maryland, USA | 1:500 | for blastn: 74%  for blastp: 63% | Goat anti-rabbit polyclonal, #A6154 (Sigma-Aldrich) | 1:20,000 |
| Dmt1 | 17-aa human C-term | Rabbit polyclonal, Alpha Diagnostic, #NRAMP22-A | 1:250 | for blastn: 83%  for blastp: 92% | Goat anti-rabbit polyclonal, #A6154 (Sigma-Aldrich) | 1:20,000 |
| HO-1 | rat,  ID J02722 | Rabbit polyclonal, Enzo Life Sciences, #ADI-OSA-150-F | 1:1000 | for blastn: 83.1%  for blastp: 92%  for blastn: 83%  for blastp: 77% | Goat anti-rabbit polyclonal, #A6154 (Sigma-Aldrich) | 1:20,000 |
| Hpx | human | Mouse polyclonal, kind gift from E. Tolosano, University of Turin, Italy | 1:1000 | for blastn: 80%  for blastp: 74% | Goat anti-mouse polyclonal, #A5278 (Sigma-Aldrich) | 1:20,000 |
| Fpn  (15) | 19-aa mouse C-term | Rabbit polyclonal, Alpha Diagnostic, #MTP11-A | 1:500 | for blastn: 90%  for blastp: 92%  for blastn: 90%  for blastp: 89% | Goat anti-rabbit polyclonal, #A6154 (Sigma-Aldrich) | 1:20,000 |
| Flvcr1 | human, ID:28982 | Rabbit polyclonal, Bioss,  #bs-12344R | 1:1000 | for blastn: 86.7%  for blastp: 84% | Goat anti-rabbit polyclonal, #A6154 (Sigma-Aldrich) | 1:20,000 |
| Hcp1 | human, 232-297-aa, internal region | Rabbit polyclonal, Santa Cruz Biotechnology, #SC-134997 | 1:500 | for blastn: 88%  for blastp: no data | Goat anti-rabbit polyclonal, #A6154 (Sigma-Aldrich) | 1:20,000 |
| L-Ft | mouse | Rabbit polyclonal, kind gift from P. Santambrogio,  Italy | 1:500 | for blastn: 76%  for blastp: 86% | Goat anti-rabbit polyclonal, #A6154 (Sigma-Aldrich) | 1:20,000 |
| Albumin | mouse  N-term | Rabbit polyclonal, Santa Cruz Biotechnology, #SC- 50536 | 1:1000 | for blastn: 75%  for blastp: 76%  for blastn: No significant similarity found  for blastp: 70% | Goat anti-rabbit polyclonal, #A6154 (SIGMA) | 1:20,000 |
| Actin | human  C-term | Goat polyclonal, Santa Cruz Biotechnology, #SC-1615 | 1:2000 | for blastn: 93%  for blastp: 100% | Donkey anti-goat polyclonal, #SC-2020 (Santa Cruz Biotechnology) | 1:20,000 |
